# Carbetocin Is More Effective in Stabilizing Hemodynamic Parameters Compared to Oxytocin During Cesarean Section

**DOI:** 10.3390/biomedicines13030685

**Published:** 2025-03-11

**Authors:** Edyta Zagrodnik, Maciej Ziętek, Tomasz Machałowski, Barbara Dołęgowska, Małgorzata Szczuko

**Affiliations:** 1 Clinical Department of Anesthesiology and Intensive Care of Adults and Children, Pomeranian Medical University in Szczecin, 72-010 Police, Poland; edyta.zagrodnik@pum.edu.pl; 2 Department of Perinatology, Obstetrics and Gynecology, Pomeranian Medical University in Szczecin, 72-010 Police, Poland; tomasz.machalowski@pum.edu.pl; 3 Department of Microbiology Immunology and Laboratory Medicine, Pomeranian Medical University in Szczecin, 71-899 Szczecin, Poland; barbara.dolegowska@pum.edu.pl; 4 Department of Human Nutrition and Metabolomics, Pomeranian Medical University in Szczecin, 71-460 Szczecin, Poland; malgorzata.szczuko@pum.edu.pl

**Keywords:** oxytocin, carbetocin, caesarean section, cardiovascular effects, hemodynamic parameters

## Abstract

**Background/Objectives**: First-line uterotonics include carbetocin and oxytocin, which act on the oxytocin receptor with varying potencies. **Methods**: In 70 pregnant Caucasian women who delivered by cesarean section, the effects of oxytocin and carbetocin on heart rate and blood pressure were compared. The pregnant women were divided into two groups: the OXY group, which received intravenous oxytocin 5 IU on an even day of the month, and the CARBE group, which received intravenous carbetocin 100 µg on an odd day of the month. Blood pressure and heart rate were measured noninvasively every 3 min from the beginning of cesarean section until the lower uterine incision, and then at 1, 2, and 3 min after the fetus and placenta were removed and the uterotonic drugs were discontinued. Subsequent measurements were taken at 3 min intervals until the end of the cesarean procedure. **Results**: After the administration of uterotonic drugs, a significant decrease in systolic blood pressure was observed only in the group receiving oxytocin at the first (*p* < 0.0001) and second minute after drug administration (*p* < 0.0001). Diastolic arterial pressure was significantly different in the study groups at the sixth minute after oxytocin and carbetocin administration (*p* = 0.004). Mean arterial pressure values were significantly different in the two study groups at the first and sixth minute after drug administration (*p* = 0.006; *p* = 0.014). With regard to heart rate, significant differences between the groups were found at 6 min after uterotonic drug administration (*p* = 0.019). **Conclusions**: Blood pressure and heart rate variability are significantly higher after oxytocin than after carbetocin administration in women delivering by cesarean section.

## 1. Introduction

In some obstetric situations, cesarean section is the only way to terminate a pregnancy. There are several risks associated with such a procedure, including obstetric hemorrhage, which is difficult to control due to disturbances in the process of uterine contraction. As a result, there is a need for pharmacological stimulation of uterine muscle contraction to reduce the risk of bleeding associated with pregnancy and labor, which accounts for approximately 6% of all deliveries [1]. Macrosomia, large parity, history of PPH (postpartum hemorrhage), and induction are risk factors for PPH [2]. Carbetocin and oxytocin in an intravenous bolus and an intramuscular combination of ergometrine and oxytocin are probably the best uterotonic agents for preventing primary postpartum haemorrhage [3]. The uterotonic drug currently recommended by the WHO for this purpose is oxytocin [4,5]. This drug acts immediately after reaching the uterine muscle and has a short half-life [6,7]. Dilating the vessels causes hypotension, tachycardia, a decrease in peripheral resistance, and an increase in cardiac minute volume, depending on the dose [8,9]. It may also cause discomfort in the form of chest pain, burning and heaviness, and headache. The short-lived effects of oxytocin sometimes require repeated doses, which may increase the risk of recurrence of the same side effects [10].

Oxytocin exerts its effects on the uterus by stimulating oxytocin receptors in the uterine muscle. It activates direct contraction of the uterine muscle via phospholipase C and the release of inositol triphosphate, leading to the release of intracellular calcium. It increases the synthesis of prostaglandins in the endometrium, which allows for the initiation of labor. Therefore, oxytocin and the synthetic analogue of carbetocin (1-deamino-1-carbo-2-tyrosine(O-methyl)-oxytocin) are the agents of choice for initiating and maintaining uterine tone [11]. The potent tonic effect of carbetocin has been demonstrated in experimental animal studies as well as in vitro studies on human myometrial slices. The potent tonic effect of carbetocin has been demonstrated in experimental animal studies as well as in vitro studies on human myometrial slices. Its potent tonic effect has been demonstrated in experimental animal studies as well as in vitro studies on human myometrial slices. Like oxytocin, carbetocin binds selectively to oxytocin receptors in uterine smooth muscle, stimulating rhythmic uterine contractions and increasing myometrial tone [12,13]. Van Dongen et al. [14] determined the maximum tolerated dose as 200 µg. The optimal therapeutic dose of this drug is between 75 and 125 µg [14]. Hunter et al. emphasize that carbetocin is a drug that has a longer tonic effect on the uterus than oxytocin, and the drug dose used is a single dose, which may be important in patients with heart disease, in whom hemodynamic stability is one of the conditions for a safe perioperative period [15]. A recent meta-analysis showed that the administration of carbetocin during caesarean section delivery in women at low risk of PPH, was associated with a lower need for additional uterine contraction drugs and, above all, with a lower need for blood transfusions and a lower fall in haemoglobin compared to women given oxytocin, with no increase in the risk of adverse effects [16]. Both of these drugs (oxytocin and carbetocin) provide changes in receptor I desensitisation levels; therefore, another aspect to also consider is the need for additional second-line uterine contraction drugs such as ergometrine, carboprost, sulprostone, or misoprostol [17]. Overall, the use of carbetocin is associated with a lower risk of PPH and the need for additional intervention [18]. Reported in the literature, the clinical effects of oxytocin and carbetocin on myometrial contraction and the occurrence of adverse effects are often inconsistent [19,20,21]. Despite the relatively long time since the introduction of the above-mentioned drugs into clinical practice, accurate data comparing the incidence and intensity of adverse cardiovascular sequelae after their use are still lacking. The available literature does not compare the effects of carbetocin and oxytocin on the haemodynamic parameters of women undergoing elective caesarean section. Analysis of such an issue will allow for a better understanding of the pharmacokinetics and pharmacodynamics of uterotonic drugs, especially in the management of patients at increased risk of hemorrhage. Additionally, the obtained data will be a practical guide to the condition of patients after the use of uterotonic drugs and the effects of administration and drug interactions.

## 2. Materials and Methods

We enrolled 70 full-term pregnant Caucasian women attending the Department of Perinatology, Obstetrics and Gynecology at the Pomeranian Medical University in Szczecin, Poland. This study was approved by the PUM Bioethics Committee (protocol code KB-0080/107/09). This study was performed from January 2019 to October 2020.

### 2.1. Study Group

This study was open to women with a singleton pregnancy who were over 37 weeks’ gestation. All women underwent an elective caesarean section. Exclusion criteria for the study included pregnant women with a previous diagnosis of heart disease, thyroid and parathyroid disease, kidney disease, diabetes, hypertension, autoimmune diseases, anemia, bronchial asthma, epilepsy, uterine bleeding, or pre-eclampsia (defined as a systolic blood pressure ≥ 140 mmHg and/or a diastolic blood pressure ≥ 90 mmHg occurring after 20 weeks’ gestation).

The women were divided into two experimental groups: OXY and CARBE. The OXY group consisted of 34 pregnant women who received 5 IU of oxytocin (Oxytocin Grindex, Joint Stock Company GRINDEX, Riga, Latvia) intravenously to induce uterine contractions. The CARBE group included 36 pregnant women who received 100 µg of carbetocin intravenously to induce uterine contraction (Pabal, Ferring Pharmaceuticals, Kiel, Germany). The groups were randomised according to odd and even monthly days. Women who delivered on an even day of the month were assigned to the OXY group, and women who delivered on an odd day of the month were assigned to the CARBE group. To standardize the groups, patients who were eligible for subarachnoid anesthesia were included in the study. The study’s drugs were administered after the neonate was delivered and the umbilical cord was clamped. The duration of intravenous administration of carbetocin and oxytocin was 20 s. The subjects gave their consent to the study; a special form was prepared. They received detailed information about the study and had the opportunity to ask questions.

### 2.2. Measured Parameters

During cesarean section, cardiac function and heart rate, blood saturation, and arterial pressure were monitored by the standard indirect method in a 3-electrode system (Monitor Infinity Delta, Draeger, Lubeck, Germany). Measurements of blood pressure, heart rate, and saturation were taken every 3 min from the beginning of the procedure until the uterine incision, and additionally at 1, 2, and 3 min after the end of uterotonic administration; the results were marked with the L1, L2, or L3 index. After this time, subsequent measurements were taken again at 3 min intervals until the end of the cesarean procedure and were assigned to the indexes L6, L9, L12 in sequence. Before the procedure, each pregnant woman received an intravenous transfusion of 500 mL of multi-electrolyte fluid and prophylaxis for pharyngitis, 30 mL of 0.3 M sodium citrate solution orally, and 200 mg of cimetidine intravenously.

### 2.3. Statistical Analysis

Statistical analysis was performed using Statistica v. 13.0 (StatSoft, Krakow, Poland). Continuous variables are presented as the arithmetic mean and standard deviation, and qualitative variables are presented as the counts and relevant percentages (fractions). The normality of the distributions of continuous variables was tested using the Shapiro–Wilk method. The two randomly selected study groups were initially compared on general characteristics using Student’s *t*-test (arithmetic means) and Pearson’s chi2 test (%). During the period from the administration of the uterine contraceptive drug to the end of the procedure, the relevant parameters were measured first every minute and then every 3 min. An ANOVA for repeated measures and Fisher’s NIR post hoc test were applied in a group x time arrangement. The significance level was set at *p* < 0.05.

## 3. Results

A comparison of the OXY and CARBE groups showed no differences in the anthropometric data of the operated pregnant women (Table 1). There were no significant differences in gestational age at which the cesarean sections were performed (*p* = 0.72). Selected laboratory parameters, such as hemoglobin, hematocrit, and Na^+^ and K^+^ ion levels, also did not differ between the study groups. Obstetric indications for cesarean section were similar between the study groups.

Regarding the parameters related to the course of anesthesia and surgery, the analysed groups were similar (Table 2). Antepartum blood pressure, pulse rate, and saturation were comparable.

**Figure 1:**
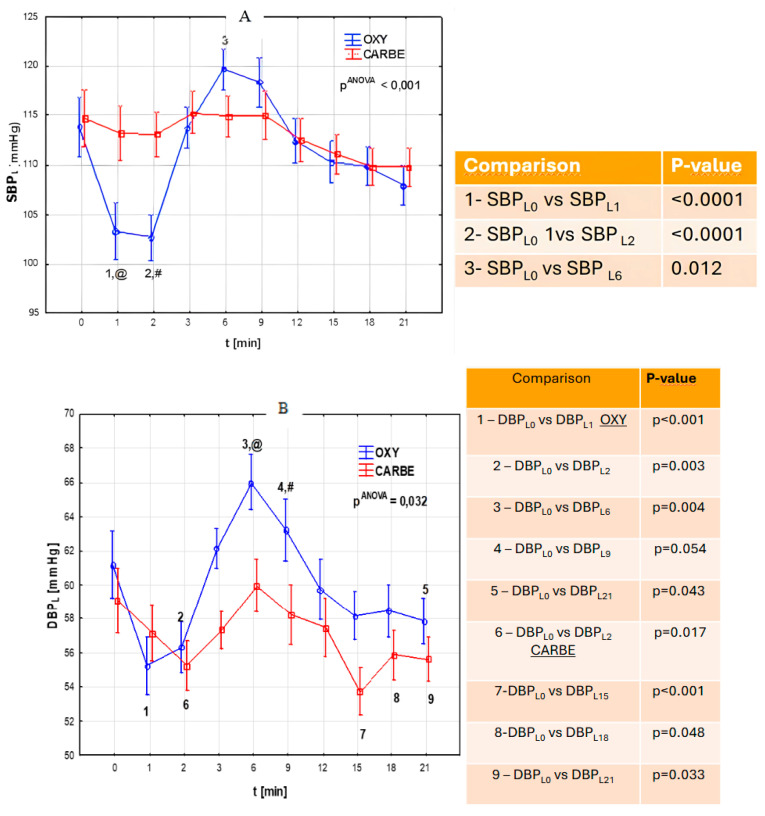

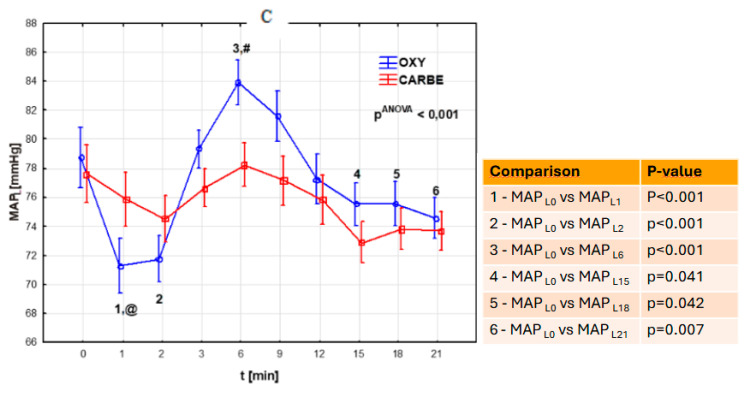
(**A**) Systolic blood pressure (SBP_L_, mmHg) during cesarean section after uterotonic drug administration in the OXY and CARBE groups. (t-time) Fisher’s NIR test results (post hoc): group OXY vs. group CARBE: @—*p* = 0.005 at time “L_1_”, #—*p* = 0.003 at time “L_2_”. (**B**) Diastolic blood pressure (DBP_L_) during cesarean section after uterotonic drug administration in the OXY and CARBE groups. (t-time) Fisher’s NIR test results-post hoc: group OXY vs. group CARBE: @—*p* = 0.007 at time “L_6_”, #—*p* = 0.026 at time “L_9_”. (**C**) Mean arterial pressure (MAP_L_) during cesarean section after uterotonic drug administration in the OXY and CARBE groups. (t-time) Fisher’s NIR test results-post hoc: group OXY vs. group CARBE: @—*p* = 0.006 at time “L_1_”, #—*p* = 0.014 at time “L_6_”.

**Figure 2:**
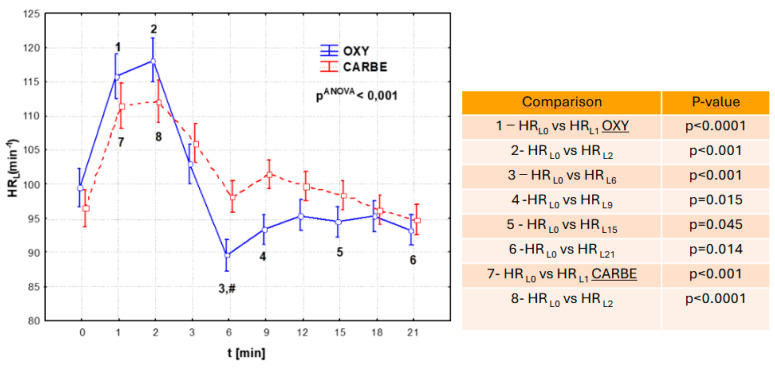
Heart rate values (HR_L_) during cesarean section after uterotonic drug administration in the OXY and CARBE groups. Fisher’s NIR test results (post hoc): group OXY vs. group CARBE: #—*p* = 0.019 at time L_6_.

The key topic of the study was to compare systolic blood pressure, diastolic blood pressure, mean arterial pressure, and heart rate after intravenous administration of oxytocin and carbetocin (Figure 1; Figure 2).

Different dynamics of changes in systolic blood pressure were observed in the studied groups. Statistically significant differences occurred only in the OXY group, in which systolic blood pressure decreased (*p* < 0.0001 in 1 min; *p* < 0.0001 in 2 min) and then increased after oxytocin administration (*p* = 0.012). In the CARBE group, the systolic blood pressure values did not differ significantly (*p* > 0.05) during the corresponding measurement periods and the course was more stable.

In the OXY group, a decrease in diastolic blood pressure was observed (*p* < 0.0001 in 1 min; *p* = 0.003 in 2 min), followed by a significant increase (after 2 min, *p* = 0.004) and then a further decrease in blood pressure (after 6 min, 0.004). In the CARBE group, there was a decrease in diastolic pressure that persisted until the end of the observation period. The study groups differed significantly in their diastolic pressure values. After oxytocin administration, higher diastolic pressure values were observed compared to CARBE during the same measurement period (Figure 1B, @—*p* = 0.007 at time “L_6_”, #—*p* = 0.026 at time “L_9_”.

Comparison of mean arterial pressure and systolic pressure showed an initial significant decrease in the OXY group (*p* < 0.001 in 1 min, *p* < 0.001 in 2 min), with a subsequent increase in MAP and a further decrease that persisted until the end of the measurement. In the CARBE group, the fluctuation of variation in the parameters studied was much smaller, and no significant changes in MAP were registered throughout the observation period.

Analysis of heart rate after the uterotonic drugs showed that after both oxytocin and carbetocin administration, there was a statistically significant increase in heart rate during the first minute after the end of drug administration. This increase was greater in the OXY group and lasted an average of one minute longer than in the CARBE group.

## 4. Discussion

The use of drugs that contract the uterine muscle and reduce the risk of obstetric hemorrhage is essential during cesarean section. Oxytocin, which has been recommended for this purpose [4,5], on the one hand, significantly reduces obstetric mortality due to obstetric hemorrhage, but, on the other hand, may contribute to myocardial ischemia [8], especially in women with coexisting heart disease. In the past, it was impossible for some patients to become pregnant (for example, with heart disease, acquired or congenital heart defects, with or without previous surgery, with cardiomyopathies or hypertension, or in whom hemodynamic stability is one of the conditions for safety in the perioperative period). In the light of medical advances in cardiology and cardiac surgery, their dream can become a reality. Studies comparing the effects of uterine muscle relaxants on the cardiovascular system are therefore warranted.

Carbetocin is a long-acting heat stable synthetic oxytocin analogue, with a half-life of about 40 min (4–10× longer than oxytocin); it binds to oxytocin receptors. The single dose of 100 µg of carbetocin can act like a 14–16 h intravenous oxytocin infusion [2]. That is the main reason for the reduction of the risk of PPH and use of additional uterotonics. The authors are uncertain; a short-infusion instead of a bolus injection may be the reason for stabilizing blood pressure and heart rate [22]. It could offer the optimal balance between a uterotonic effect and a reduction of cardiovascular side effects whilst minimising the risk of PPH [2,16]. One of the first studies comparing the effects of oxytocin and carbetocin on selected hemodynamic parameters in pregnant women during cesarean section was conducted by Moertl et al. [23]. The authors, comparing changes in directly measured systolic, diastolic, and mean arterial pressure, found the greatest decreases 30–40 s after the administration of both drugs. These changes were accompanied by an increase in heart rate. After the administration of oxytocin and carbetocin, the authors of the study recorded decreases in systolic pressure, diastolic pressure, and mean arterial pressure. The changes in pressure levels analysed by the authors were not statistically or clinically significant, and pressure levels returned to near baseline levels after approximately 500 s.

Similarly, in a study by Rosseland et al. [24], who compared changes in systolic pressure, diastolic pressure, mean arterial pressure, and heart rate between groups, the greatest decrease in mean arterial pressure was observed after oxytocin and carbetocin administration 80 s after oxytocin administration and 63 s after carbetocin administration, with the decrease in MAP greater after oxytocin administration. Differences ceased to be significant in the study groups at 2.5 min after the time of drug administration. Discrepancies in systolic blood pressure decreased between the study groups within 5 min of drug administration and were unremarkable after one hour. The results obtained in our study differ from those published by Moertl. In the present study, a decrease in systolic blood pressure was observed only in the group of women who received oxytocin. The recorded decrease was smaller than in the Moertl study [23]. No significant changes in systolic blood pressure values were observed after carbetocin in the analyzed time interval. The CARBE group also registered a smaller range of variation in diastolic and mean arterial pressures.

In a study presented by Bahr et al., comparing changes in hemodynamic parameters after oxytocin and carbetocin in patients undergoing cesarean section, a statistically significant decrease in SBP (at 1, 5 and 15 min) and MAP (at 1 min) was recorded in the group of patients receiving oxytocin. Similar results were obtained in our own work, where a decrease in SBP was observed only in the OXY group [25]. The described changes in values of systolic blood pressure, diastolic blood pressure, and mean arterial pressure after the use of drugs modulating myometrial contractility may be related to the duration of intravenous administration of drugs of this group. The reports in the literature that confirm the relationship between the duration of intravenous administration of uterotonic drugs and the hemodynamic stability of cardiovascular parameters refer only to oxytocin [7,17,19]. There are no studies in the available literature on the specific duration of carbetocin administration and the resulting changes in cardiovascular parameters.

In our study, the time for intravenous injection of the uterotonic was 20 s, while in the cited Moertl study, it was half as long [23]. The two times longer time of intravenous injection of uterotonics in our own study and the observed smaller range of fluctuations in pressure values allow us to assume that greater hemodynamic stability of parturients was achieved after the use of drugs from this group with such a specific procedure. Moertl et al., who measured blood pressure directly, recorded the first drop in systolic blood pressure 30–40 s after the use of oxytocin and carbetocin [23]. In our study, a non-invasive method of blood pressure measurement was used and, therefore, the first measurements documenting a statistically significant drop in blood pressure were not obtained until one minute after the application of the uterotonic drug. Earlier blood pressure fluctuations, even if they occurred, may have gone unnoticed in this situation, which may explain the results obtained. Regarding heart rate changes, comparable heart rate fluctuations were observed in the first three minutes after the application of oxytocin and carbetocin in both study groups. A statistically significant difference between the groups was registered after the sixth minute after the application of uterotonic drugs, with higher heart rate values in the CARBE group, which remained clinically insignificant. The reported fluctuation of heart rate changes after oxytocin and carbetocin differs from the study by Bahr et al. [25], where a significant increase in HR was recorded in the first minute in the group receiving oxytocin. However, the greater increase in HR and the greater decrease in SBP, DBP, and MAP in the oxytocin group were statistically significant only at different time points.

Our results are partially consistent with those published by Moertl et al. [23]. These authors found higher heart rate values after carbetocin administration and then observed a slow, asymptomatic return of heart function to a value close to the initial one. In the cited study, the range of change in heart rate values was greater after oxytocin administration, and the subsequent decrease reached a value up to 7% lower than the initial magnitude. These results are similar to those of the present study, in which a greater dynamic range of heart rate changes was observed after oxytocin administration, including decreases in heart rate values up to 10% below baseline and less fluctuation in heart rate changes after carbetocin administration. Similarly, in a study reported by Rosseland et al. [24], where heart rate and cardiac output increased after both oxytocin and carbetocin administration, interestingly, the increase was also noted in the placebo group, with stroke volume increasing after oxytocin and carbetocin and unchanged in the placebo group.

In the results obtained, it is noteworthy that after the administration of oxytocin, the increase in heart rate was accompanied by a significant decrease in systolic blood pressure, diastolic blood pressure, and mean arterial pressure, similar to the work of Mahmoud Hussein Bahr. After the administration of carbetocin, a decrease was observed in the diastolic pressure, with no significant changes in systolic pressure and mean arterial pressure. The moment of significant increase in systolic blood pressure, diastolic blood pressure, and mean arterial pressure 6 min after oxytocin administration, accompanied by a decrease in cardiac function, noted in our study, may be of clinical significance, especially in pregnant women with coexisting heart disease.

In a study by Maria Egeland Bekkenes et al. [26], comparing the effects of oxytocin and carbetocin on the release of myocardial biomarkers (troponin I and T), QT interval prolongation, and ST segment depression, a significantly higher level of troponin I was observed in the oxytocin group at 4 and 10 h after cesarean section. In both groups, the increase in troponin I and T was most pronounced at 10 h after cesarean section. The QT segment was prolonged in both groups at 9 min after drug administration, with no statistically significant differences in ST segment depression between the study groups. The need for repeated administration of the drug in selected clinical situations, as well as the dosage size of the drug and the duration of intravenous administration, may further disrupt the existing cardiovascular balance.

Although the cost of carbetocin is high, given the significant benefits it provides in terms of effective uterine contraction, lack of disturbance of hemodynamic parameters, and reduced risk of postpartum hemorrhage, it ultimately seems that carbetocin is the most beneficial drug clinically [27], especially when used in patients undergoing elective cesarean section [28]. In a retrospective cohort study by Terblanche NC et al., the risk of major postpartum hemorrhage in women who received carbetocin was lower than in those who received oxytocin during cesarean section, which was in accordance with the results of other investigators [29].

The above study has its limitations. Given that cesarean section is a common surgical procedure in maternity wards, the study should include a much larger group of surveyed women giving birth in multiple centers. Also, the emotional status and stress levels of the women studied were not taken into account, which could temporarily affect the heart rate and blood pressure parameters of the operated women.

## 5. Conclusions

In conclusion, the results of the present study show that haemodynamic stability is improved after administration of carbetocin compared with oxytocin in women undergoing caesarean section. These results suggest that carbetocin may be the preferred uterotonic agent for pregnant women who require optimal haemodynamic stability. The results of the above study from the point of view of the vital parameters of the operated women, such as blood pressure and heart rate, prove that carbetocin is a better drug than oxytocin for use during caesarean section.

## Figures and Tables

**Table 1:** Anthropometric characteristics of pregnant women in the OXY and CARBE groups.

Parameter	Group OXY n = 34			Group CARBE n = 36			*p*-Value
	Mean ± SD	Min.–Max. Value	Median	Mean ± SD	Min.–Max. Value	Median	
Age (years)	29.7 ± 5.3	20–39	30.0	29.3 ± 4.9	20–46	29.5	0.72
Gestational age (weeks)	38.5 ± 1.4	37–41	39.0	38.9 ± 0.9	37–40	39.0	0.16
Body weight (kg)	76.5 ± 9.6	55–97	76.5	79.8 ± 12.7	57–110	78.0	0.23
Height (cm)	165.3 ± 5.0	156–176	165.5	166.0 ± 4.1	158–182	164.5	0.59
BMI (kg/m^2^)	28.0 ± 3.4	22.6–37.8	28.1	28.9 ± 4.1	20.1–37.0	28.3	0.32

*p*-value for Student’s *t*-test, *p*—statistical significance, SD—standard deviation.

**Table 2:** Anesthetic and cesarean section procedure course in the OXY and CARBE groups.

Variable	Group OXY n = 34 Mean ± SD	Group CARBE n = 36 Mean ± SD	*p*-Value
Dose of Marcaine Spinal Heavy (mg)	12.4 ± 1.5	12.8 ± 1.7	0.415
Time from start of surgical procedure to umbilical cord clamping (min)	6.1 ± 3.0	6.2 ± 4.0	0.955
Neonatal birth weight (g)	3261.2 ± 5.0	3455.6 ± 5.0	0.124
Total volume of intravenous fluids (mL)	784.6 ± 97.6	803.6 ± 85.4	0.274

*p*-value for Student’s *t*-test, *p*—statistical significance, SD—standard deviation.

## Data Availability

Data are available on request from the first author.
